# Sharp‐wave ripple features in macaques depend on behavioral state and cell‐type specific firing

**DOI:** 10.1002/hipo.23046

**Published:** 2018-11-22

**Authors:** Ahmed T. Hussin, Timothy K. Leonard, Kari L. Hoffman

**Affiliations:** ^1^ Department of Biology, Centre for Vision Research York University Toronto Ontario Canada; ^2^ Department of Psychology, Centre for Vision Research York University Toronto Ontario Canada; ^3^ Department of Psychology, Center for Integrative and Cognitive Neuroscience, Vanderbilt Vision Research Center Vanderbilt Brain Institute, Vanderbilt University Nashville Tennessee

**Keywords:** bursting, electrophysiology, hippocampus, local field potential, memory, nonhuman primate

## Abstract

Sharp‐wave ripples (SWRs) are spontaneous, synchronized neural population events in the hippocampus widely thought to play a role in memory consolidation and retrieval. They occur predominantly in sleep and quiet immobility, and in primates, they also appear during active visual exploration. Typical measures of SWRs in behaving rats include changes in the rate of occurrence, or in the incidence of specific neural ensemble activity contained within the categorical SWR event. Much less is known about the relevance of spatiotemporal SWR features, though they may index underlying activity of specific cell types including ensemble‐specific internally generated sequences. Furthermore, changes in SWR features during active exploratory states are unknown. In this study, we recorded hippocampal local‐field potentials and single‐units during periods of quiescence and as macaques performed a memory‐guided visual search task. We observed that (a) ripples during quiescence have greater amplitudes and larger postripple waves (PRW) compared to those in task epochs, and (b) during “remembered” trials, ripples have larger amplitudes than during “forgotten” trials, with no change in duration or PRWs. We further found that spiking activity influences SWR features as a function of cell type and ripple timing. As expected, larger ripple amplitudes were associated with putative pyramidal or putative basket interneuron (IN) activity, even when the spikes in question exceed the duration of the ripple. In contrast, the PRW was attenuated with activity from low firing rate cells and enhanced with activity from high firing rate cells, with putative IN spikes during ripples leading to the most prominent PRW peaks. The selective changes in SWR features as a function of time window, cell type, and cognitive/vigilance states suggest that this mesoscopic field event can offer additional information about the local network and animal's state than would be appreciated from SWR event rates alone.

## 1 INTRODUCTION

The sharp‐wave ripple (SWR) is a highly synchronized neural population event in the hippocampus that is widely thought to support memory. Ripples are typically detected in the hippocampal local field potential (LFP) arising from synaptic and spiking activity in local neuronal populations (Buzsáki, 2015; Schomburg, Anastassiou, Buzsáki, & Koch, 2012). Ripples occur most frequently during non‐REM sleep, where they are important for memory consolidation (Ego‐Stengel & Wilson, 2010; Girardeau, Benchenane, Wiener, Buzsáki, & Zugaro, 2009; Nokia, Mikkonen, Penttonen, & Wikgren, 2012), and less frequently during waking, where they appear to be important for memory‐based decision‐making (Jadhav, Kemere, German, & Frank, 2012; Leonard & Hoffman, 2017; Wu, Haggerty, Kemere, & Ji, 2017). During ripples, firing sequences observed during earlier waking periods are replayed among local populations within the hippocampus (Csicsvari, O'Neill, Allen, & Senior, 2007; Diba & Buzsáki, 2007; Foster & Wilson, 2006; Ji & Wilson, 2007; Lee & Wilson, 2002; Nadásdy, Hirase, Czurko, Csicsvari, & Buzsáki, 1999), and at distant neocortical (Ji & Wilson, 2007; Peyrache, Khamassi, Benchenane, Wiener, & Battaglia, 2009; Qin, McNaughton, Skaggs, & Barnes, 1997) and subcortical (Gomperts, Kloosterman, & Wilson, 2015; Pennartz et al., 2004) sites. This “replay” phenomenon is thought to involve the synaptic modifications of relevant neural ensembles, supporting theories about the role of ripples in memory consolidation (Buzsáki, 2015; Carr, Jadhav, & Frank, 2011; Girardeau & Zugaro, 2011; Roumis & Frank, 2015; Sadowski, Jones, & Mellor, 2011). When ripples are disrupted, memory is impaired, suggesting a causal role for the neural activity occurring during ripples in memory formation (Ego‐Stengel & Wilson, 2010; Girardeau et al., 2009; Jadhav et al., 2012; Nokia et al., 2012).

Because the ripple mean field potential (or ripple‐LFP) arises from the synchronous activity of neuronal ensembles thought to be critical for memory formation, it is important to understand how the activity of local cell populations shapes the ripple‐LFP. Following a ripple, a brief period of hyperpolarization ensues where spikes are suppressed (English et al., 2014; Hulse, Moreaux, Lubenov, & Siapas, 2016). This period, which is observed in the ripple‐LFP as a positive polarity deflection (or postripple wave, PRW), may be additionally valuable in decoding local circuit activity immediately prior to and during the ripple. In general, neuronal firing rate and/or phase‐locked firing are associated with high frequency (>50 Hz) LFP (Anastassiou, Perin, Buzsáki, Markram, & Koch, 2015; Belluscio, Mizuseki, Schmidt, Kempter, & Buzsáki, 2012; Montefusco‐Siegmund, Leonard, & Hoffman, 2017; Ray, Crone, Niebur, Franaszczuk, & Hsiao, 2008; Scheffer‐Teixeira, Belchior, Leão, Ribeiro, & Tort, 2013). More specifically, the spatiotemporal features of the ripple‐LFP can vary according to the specific neural ensembles active during the ripple. This relationship has been used to decode replay spiking content based on the similarity of ripple features alone (Taxidis, Anastassiou, Diba, & Koch, 2015).

The relationship between spiking activity and ripple features becomes more complicated when considering different vigilance states and corresponding changes in neuromodulatory tone (Atherton, Dupret, & Mellor, 2015). Despite numerous reports measuring ripple occurrence, few studies have investigated how ripple‐LFP features vary with learning. In one study, ripple amplitude was observed to be greater during sleep when followed by learning (Eschenko, Ramadan, Molle, Born, & Sara, 2008). Sharp‐wave amplitude during sleep has also been shown to be greater than in waking (Buzsáki, 2015; O'Neill, Senior, & Csicsvari, 2006). Other investigations into the variance in ripple amplitude found a positive correlation with spiking activity of a cell class in the cingulate cortex, suggesting that ripple‐LFP features can predict spiking activity not only locally in the hippocampus but also even in distal neocortical areas (Wang & Ikemoto, 2016).

Characterization of cell‐type specific firing during ripples and their relation to SWR features is especially lacking in behaving primates where ripple physiology seems to be generally complementary to that observed in rats and mice (Bragin et al., 1999; Skaggs et al., 2007; Le Van Quyen et al., 2008, Logothetis et al., 2012; Leonard et al., 2015; Leonard & Hoffman, 2017). Despite the many similarities, a key difference is that ripples occur not only during awake immobility in primates but also during active visual exploration (Leonard et al., 2015; Leonard & Hoffman, 2017). To date, the only features measured during exploratory SWRs were their rate of occurrence and peak frequency, which did not differ by state.

In this study, we examined how three ripple‐LFP features vary across waking states and as a function of learning, in addition to their modulation by spiking activity (single‐unit activity, SUA). We found that ripple and PRW amplitude in macaques are greater during quiescence than waking and that on remembered trials in a visual‐search memory task, ripple amplitude is increased, with no change to duration or PRWs. We also describe the SWR modulation by cell types, classified by burstiness and firing rate, finding that low‐firing rate cells (putative principal cells) are associated with enhanced ripple amplitude and attenuated postripple amplitude, whereas high‐firing bursting and nonbursting cell types (putative basket interneurons) are associated with enhanced ripple and PRW amplitudes.

## 2 MATERIALS AND METHODS

### 2.1 Subjects and experimental design

**Figure 1:**
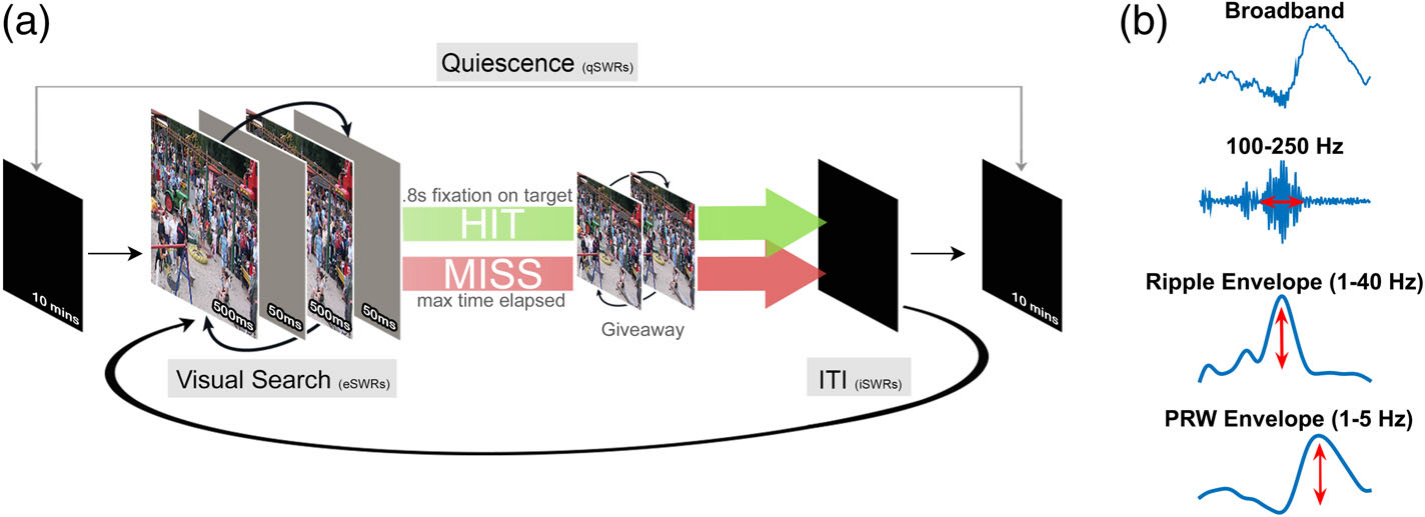
Experimental design of memory‐guided visual search task and signal processing. (a) An original and modified scene is presented in alternation, interleaved with a brief grey mask, requiring an effortful search to detect the changing target. A trial ends with a 0.8 s fixation on the target for which a fluid reward is delivered (HIT), or when the maximum trial time is reached (MISS). A “giveaway” then follows in which the two scenes are displayed without a mask, revealing the target location. A trial ends with a black screen inter‐trial‐interval (ITI) of 2–22 s before the next trial is presented. During daily recording sessions, scenes are presented in blocks of 30 and the task is bookended with two rest periods (quiescence; ≥10 min) where a black screen is presented and animals sleep. See *Materials and Methods* for more details. (b) The broadband LFP signal is bandpass filtered in the ripple band (100–250 Hz), *z*‐scored, rectified and then low pass filtered (1–40 Hz) to create the ripple envelope whose maximum value represents the ripple amplitude. The PRW envelope is a low pass filter (1–5 Hz) of the broadband signal and its peak represents the PRW amplitude

Two adult female macaques (*Macaca mulatta*, named LU and LE) completed a visual target‐detection task that requires hippocampal function in primates (Chau, Murphy, Rosenbaum, Ryan, & Hoffman, 2011), during daily recording sessions (this data set was used in Leonard et al. (2015) and Leonard and Hoffman (2017). The flicker change‐detection task [previously described in Leonard et al. (2015) and Leonard and Hoffman (2017)] required the animals to find and select a target object from nontargets in unique visual scenes for fluid reward (Figure 1a). Selection of a scene‐unique target object was accomplished by holding gaze in the target region for a prolonged (≥800 ms) duration. The target object was defined as a changing item in a natural scene image, where the original and changed images were presented in alternation, each lasting 500 ms, with a brief grey‐screen (50 ms) shown between image presentations. Displayed this way, detection of the changing part of the scene requires an effortful search in humans and macaques (Chau et al., 2011). An inter‐trial interval (ITI) of 2–20 s followed each trial. The daily sessions began and ended with a period of at least 10 min when no stimulus was presented within the darkened booth and animals were allowed to sleep or sit quietly (quiescent period). Eye movements were recorded using video‐based eye tracking (iViewX Hi‐Speed Primate remote infrared eye tracker). All experimental protocols were conducted with approval from the local ethics and animal care authorities (Animal Care Committee, Canadian Council on Animal Care).

### 2.2 Electrophysiological recordings

Both animals were chronically implanted with independently moveable platinum/tungsten multicore tetrodes (96 μm outer diameter; Thomas Recordings) lowered into hippocampal CA3/DG regions. Animal LE had a 9‐tetrode bundle centered at AP +11 mm verified postimplant with MRI. For this study we analyzed activity from the 4/9 tetrodes placed to optimize ripple and unit responses; these tetrodes were separated by <600 μm in the bundle. Animal LU had 8 tetrodes divided into two bundles: one at AP +11 mm and the other at AP +8 mm verified with postoperative CT co‐registration to MRI. Based on ripple and unit activity we analyzed 3 tetrodes from each bundle, with separation <500 μm in the bundles). LFPs were digitally sampled at 32 kHz using a Digital Lynx acquisition system (Neuralynx) and filtered between 0.5 Hz and 2 kHz. Single‐unit activity was sampled at 32 kHz and filtered between 600 Hz and 6 kHz, recording the waveform for 1 ms around a threshold triggered spike events. Single units were isolated using MClust based on wave‐shape principle components, energy and peak/valley across channels. Only well‐isolated cells were included, based on <1% interspike intervals (ISIs) within 2 ms and cross‐correlograms between bursting cell pairs that had to be free of burst‐latency peaks (asymmetric, <10 ms peak that could indicate the erroneous splitting of one CS unit into two; Harris, Henze, Csicsvari, Hirase, & Buzsáki, 2000). Units were classified as putative principal units (PR) if they had a burst firing mode (ISI mode peak, <10 ms, comprising ≥10% of ISIs) and under <1 Hz overall firing rate. Units were classified as putative interneurons (IN) if they had no burst firing mode (>10 ms ISI) and a firing rate >1 Hz. The remaining two possible categories were the burst firing mode with >1 Hz firing rate (BHF), and nonburst firing mode with <1 Hz spiking rate (NBLF). Waveshape parameters such as spike width and peak‐trough asymmetry can vary as a function of recording location relative to the cell body and not only by cell type (Henze et al., 2000, figure 8), therefore these waveshape measures were not used for cell type classification in this study.

### 2.3 SWR detection and feature estimation

SWR events were detected using the tetrode channel with the most visibly apparent ripple activity. The LFP signal was bandpass filtered (100–250 Hz), transformed into z‐scores, rectified and then low pass filtered (1–40 Hz). Ripple events were defined as threshold crossings 3 *SD*s above the mean, with a minimum duration of 50 ms beginning and ending at 1 *SD*. This time period also defined the ripple duration. SWR amplitude was defined as the maximum peak of the ripple envelope (*z*‐score). The amplitude of the PRW was defined as the maximum peak (*z*‐score) of a narrower lowpass filter (1–5 Hz, Figure 1b). SWR amplitude, duration and PRW amplitude values were then normalized per tetrode for each animal. The use of the z transformation preserved the shape of the distributions (i.e., the relative magnitude differences from the mean) that would be lost with percentile/rank order, while ensuring an even scaling across tetrodes in case of overall differences in ripple amplitude. Each feature of the SWR (ripple duration, amplitude, and PRW amplitude) was then compared across different states and task epochs.

### 2.4 SWR features across behavioral epochs

SWRs were clustered depending on time of occurrence into three behavioral epochs; quiescence (10 min dark‐booth time period at the beginning and end of every session, qSWR), ITI (2–22 s interval between scene presentations representing quiet waking “inactive” states, iSWR), and exploratory search (during “active” visual search, eSWR). We excluded search ripples that occurred while the monkey fixated off‐screen, and during search trials where the monkey spent >40% of trial time fixating off‐screen. Task SWRs were further clustered by stimulus repetition into novel (scene repetition number = 0) and repeated trials (scene repetition number > 0), and repeated trial ripples were further clustered into ripples occurring during trials where the target was successfully found (HIT), and when the target was not (MISS).

### 2.5 Statistical analysis

Ripple features across waking state and task epochs were compared using the Wilcoxon rank‐sum test and the Kolmogorov–Smirnov (K–S) test. For the single‐unit and ripple‐LFP waveform analysis, a Kruskal–Wallis test was conducted with a Bonferroni correction for multiple comparisons.

## 3 RESULTS

Based on SWR clustering described above, we detected 2,526 qSWRs (LU = 1866, LE = 660), 536 iSWRs (LU = 340, LE = 196), and 664 eSWRs (LU = 462, LE = 202) from a total of 77 recording sessions (LU = 45, LE = 32). Based on unit clustering described earlier, we recorded from a total of 509 units: 242 PRs, 133 NBLFs, 48 BHFs and 86 INs.

### 3.1 SWR features across states

**Figure 2:**
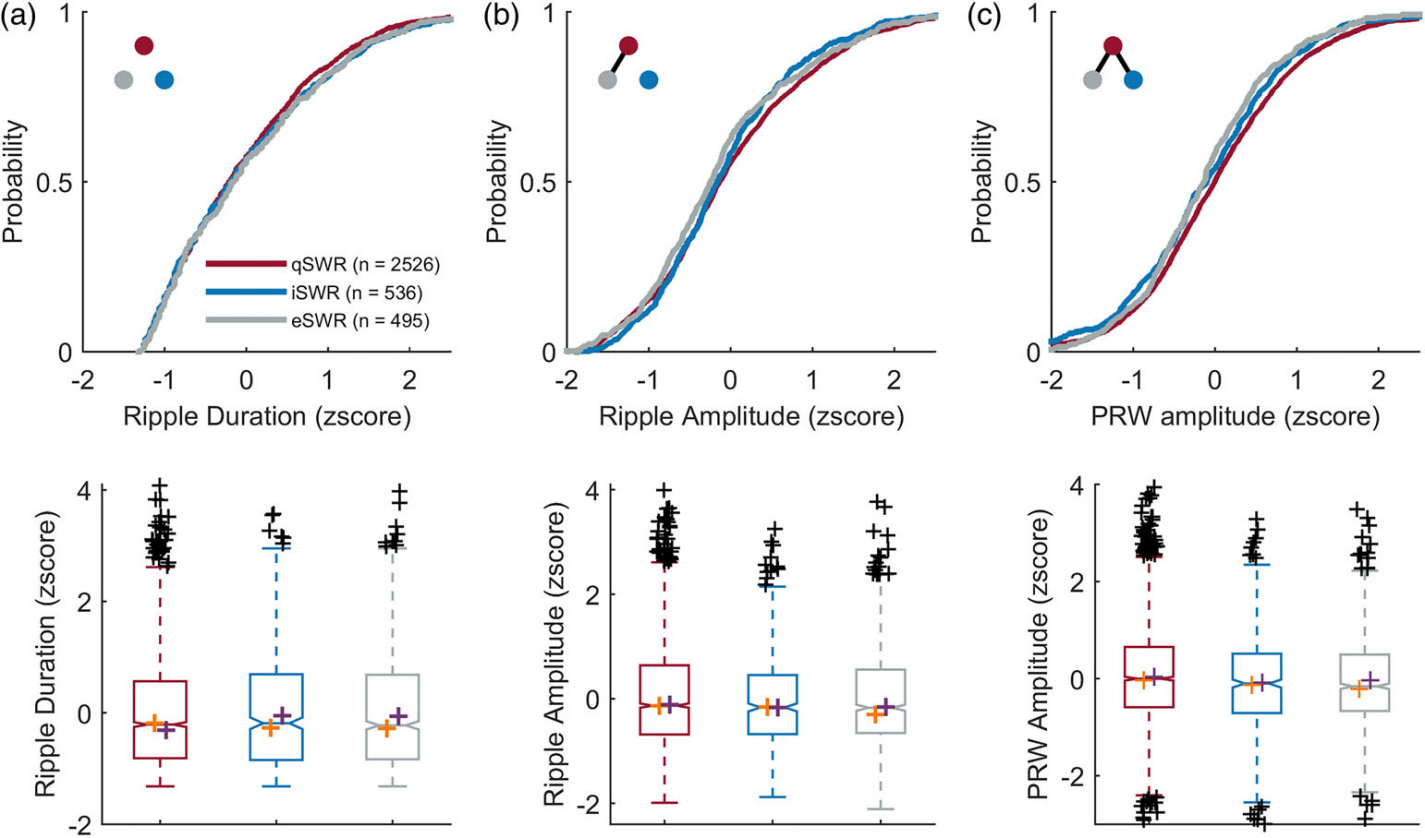
Ripple and PRW amplitudes are greater during qSWRs than iSWRs and eSWRs. *Top*: cumulative probability distribution. Blackline connecting dots in the top left inset of top panels indicates *p* < .05 between groups, as represented by dot color. *Bottom*: boxplots of corresponding distributions above with median values for each animal plotted in orange (for LU) and purple (for LE) crosses, for SWR duration (a), amplitude (b) and PRW amplitude (c) across qSWRs (*n* = 2,526), iSWRs (*n* = 536), and eSWRs (*n* = 495)

We first examined SWR duration, amplitude and PRW amplitude across the different states (qSWR, iSWR, and eSWR; Figure 2). SWR duration was not different across states (rank sum and K‐S test *p* > .5; Figure 2a), whereas ripple amplitude was greater during qSWRs compared to eSWRs (rank sum *z* = 2.48, *p* = 1.31 × 10^−2^; K–S *d* = 8.0 × 10^−2^, *p* = 8.4 × 10^−3^, Figure 2b), and PRW amplitude was greater in qSWRs compared to iSWRs (rank sum: *z* = 2.79, *p* = 5.30 × 10^−3^, K–S *d* = 7.0 × 10^−2^, *p* = 2.3 × 10^−2^) and eSWRs (rank‐sum: *z* = 3.33, *p* = 8.63 × 10^−4^, K–S *d* = 9.1 × 10^−2^, *p* = 2.0 × 10^−3^, Figure 2c).

### 3.2 SWR features during recognition memory task

**Figure 3:**
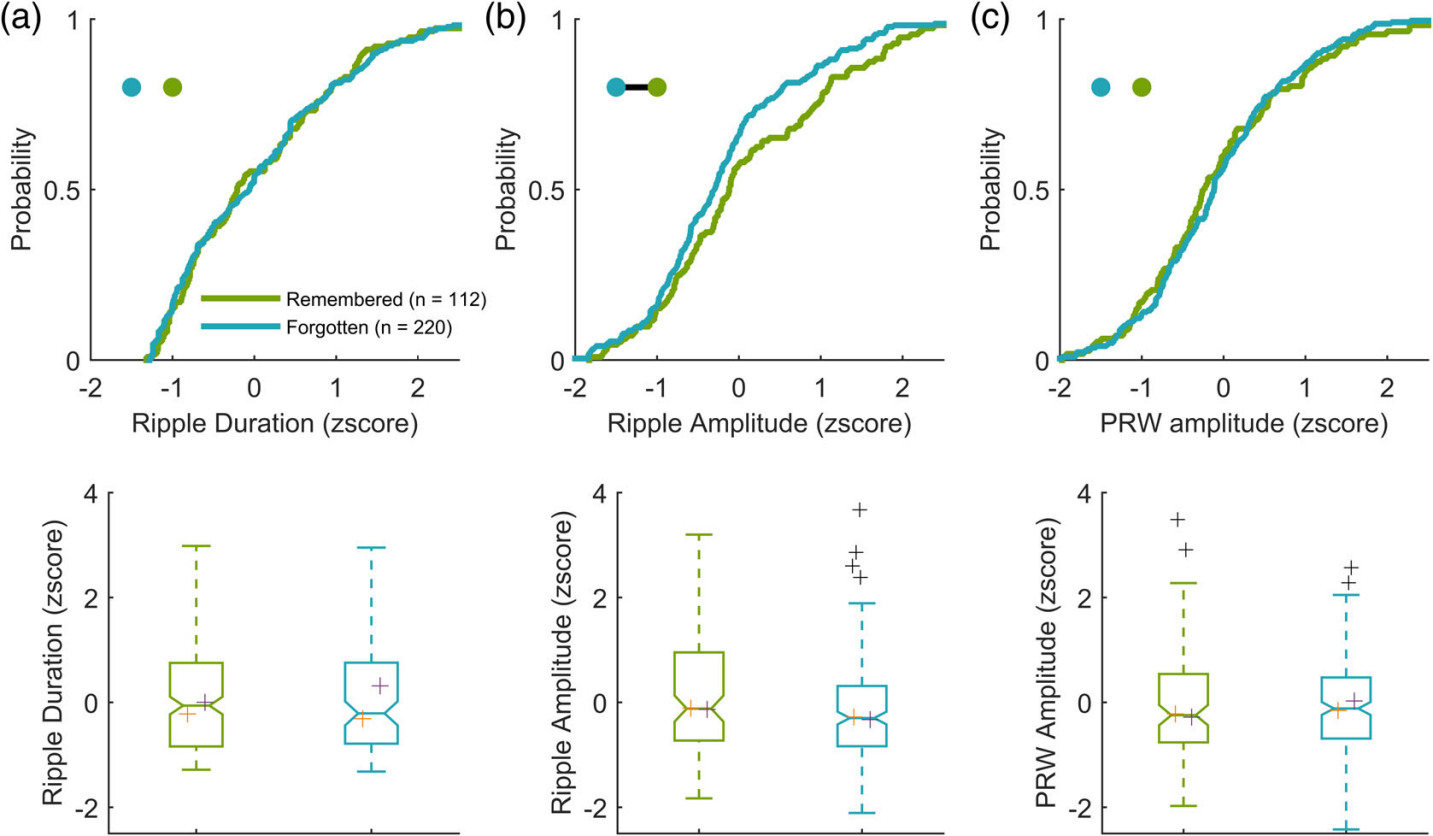
SWR amplitude, but not duration or PRW amplitude, is greater during remembered trials during the goal‐directed visual search. *Top*: cumulative probability distribution. Blackline connecting dots in the top left inset of top panels indicates *p* < .05 between groups, as represented by dot color. *Bottom*: boxplots of corresponding distributions above. Median values for each animal are plotted in orange (LU) and purple (LE) crosses for SWR duration (a), amplitude (b), and PRW amplitude (c) during remembered (*n* = 220) and forgotten (*n* = 112) trials

Previously, we found that ripples occur more frequently and closer to a visual target with learning (Leonard & Hoffman, 2017). We therefore asked whether ripples that occur on repeated trials are different in duration or amplitude. First, we examined whether features vary by scene repetition by splitting ripples into novel (repetitions = 0) and repeated (repetitions >0), but found no differences in ripple duration, amplitude, or PRW amplitude between novel and repeated trials (rank sum and K–S tests *p* > .05). Next, we split repeated trials into trials where the target was successfully found (indicating memory for the target location), and not found (indicating forgetting). Ripple duration (Figure 3a) and PRW amplitude (Figure 3c) were not different between remembered and forgotten trials (rank sum and K–S tests *p* > .5). During remembered trials (*n* = 112) ripple amplitude was larger than forgotten trials (*n* = 220) (rank sum *z* = 2.11, *p* = 3.5 × 10^−2^, K–S test *d* = 0.16, *p* = 3.6 × 10^−2^, Figure 3b). Because we had observed a greater ripple amplitude during quiescence compared to search, we compared ripple amplitude on remembered trials and quiescence but found no difference (rank sum: *z* = 0.59, *p* = .55, K–S *d* = 8.5 × 10^−2^, *p* = .41).

### 3.3 SUA analysis

**Figure 4:**
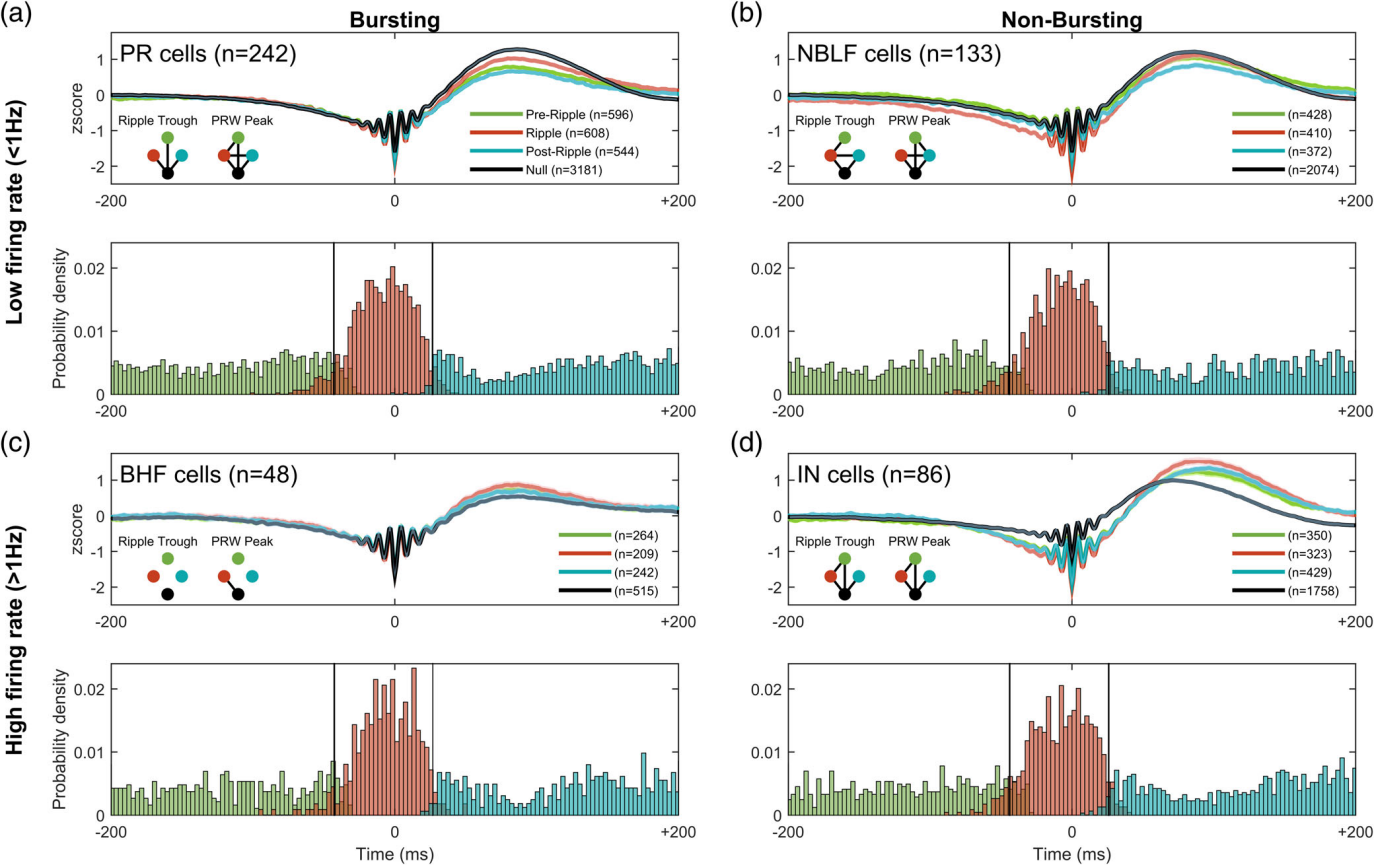
Ripple waveform varies by cell‐type activity and spike timing relative to the SWR event. (A) *Top*: mean ± 95% confidence intervals of broadband SWRs grouped by putative principal cells' spike‐timing into preripple, ripple, PRW, and null. Blackline connecting dots in the lower left inset of top panel indicates *p* < .05 between groups, as represented by dot color, for respective ripple feature. *Bottom*: probability density histogram of spike counts in a ± 200 ms window centered around the maximum ripple amplitude for putative principal units. The ripple window membership (pre, ripple, post) is indicated by the color of the histogram bar. (b) as in (a) but for nonbursting low‐firing rate units; (c) for bursting high‐firing rate, and (d) for putative interneuron units

Next, we examined local cell‐type specific firing underlying ripples. Spikes occurring in a 400 ms time window centered around the peak of the ripple envelope were clustered based on spike‐timing relative to the ripple event. Spikes were clustered into *preripple*, if they occurred before the ripple, *ripple*; if they occurred during the ripple or *postripple*; if they occurred after the ripple (during the PRW). For each functional‐unit type, the average ripple‐LFP waveform was calculated based on the window of spike‐times aligned to ripple peak (Figure 4). Also calculated for each unit is the average ripple waveform where no spikes were observed (Null), and below each waveform plot is the normalized spike count distribution for each unit class in the ripple window clustered by spike‐timing (preripple, ripple, and postripple).

### 3.4 SUA effects on ripple trough

We observed different effects on the magnitude of the ripple trough (defined as nearest trough to ripple peak) based on spike‐time occurrence for PR (Figure 4a, H[3] = 78.65, *p* = 5.99 × 10^−17^), NBLF (Figure 4b, H[3] = 141.10, *p* = 2.2 × 10^−30^), IN (Figure 4d, H[3] = 360.89, *p* = 6.53 × 10^−78^), but not BHF cells (Figure 4c, H[3] = 5.19, *p* = .16). In PR cells, spiking in any of the time windows (preripple, ripple, or postripple) was associated with a larger trough compared to no spikes (*p* < .05, Bonferroni post hoc test; mean LE‐PR‐ripple = −1.79z, LE‐PR‐null = −1.59, LU‐PR‐ripple = −2.74z, LU‐PR‐null = −1.58z). In NBLF cells, spiking in the ripple window was associated with a greater trough compared to spiking in preripple and postripple windows, as well as no spiking (*p* < .05, Bonferroni post hoc; mean LE‐NBLF‐ripple = −1.92z, LE‐NBLF‐null = −1.61; LU‐NBLF‐ripple = −2.75z, LU‐NBLF‐null = −1.54z). In IN cells, a similar pattern followed whereby ripple spikes were associated with a larger trough compared to preripple and no spikes (*p* < .05, Bonferroni post hoc; LE‐INT‐ripple = −1.73z, LE‐INT‐null = −1.59z; LU‐INT‐ripple = −2.3z, LU‐INT‐null = −1.04z). Interestingly, for nonbursting low firing rate cells that fired during the ripple, the LFP showed slow negative deflections in the ~200 ms leading up to the ripple event.

### 3.5 SUA effects on the PRW

The peak magnitude of the PRW in the broadband signal varied according to spike‐time occurrence and as a function of cell type, among PR (H[3] = 693.67, *p* = 4.94 × 10^−150^), NBLF (H[3] = 150.59, *p* = 1.96 × 10^−32^), IN (H[3] = 25.10, *p* = 1.47 × 10^−5^), and BHF cells (H[3] = 11.90, *p* = 7.7 × 10^−3^). The spiking of low firing‐rate cells (PR and NBLF cells, Figure 4a,b) during the ripple window was associated with smaller peaks compared to null spiking (*p* < .05, Bonferroni post hoc), whereas the opposite effect was seen with high firing‐rate cells (BHF and IN cells, Figure 4c,d) where spiking was associated with a larger PRW (*p* < .05, Bonferroni post hoc). For low firing‐rate cells (Figure 4a,b), spiking during the postripple window resulted in the smallest peak (*p* < .05, Bonferroni post hoc). For high firing‐rate cells (Figure 4c,d), spiking during the ripple was associated with the largest peaks (*p* < .05, Bonferroni post hoc). The heightened modulation for both peaks and troughs found for the IN group suggests a stronger overall ripple amplitude, measured explicitly below.

### 3.6 SUA effects on the amplitude of the ripple envelope

**Figure 5:**
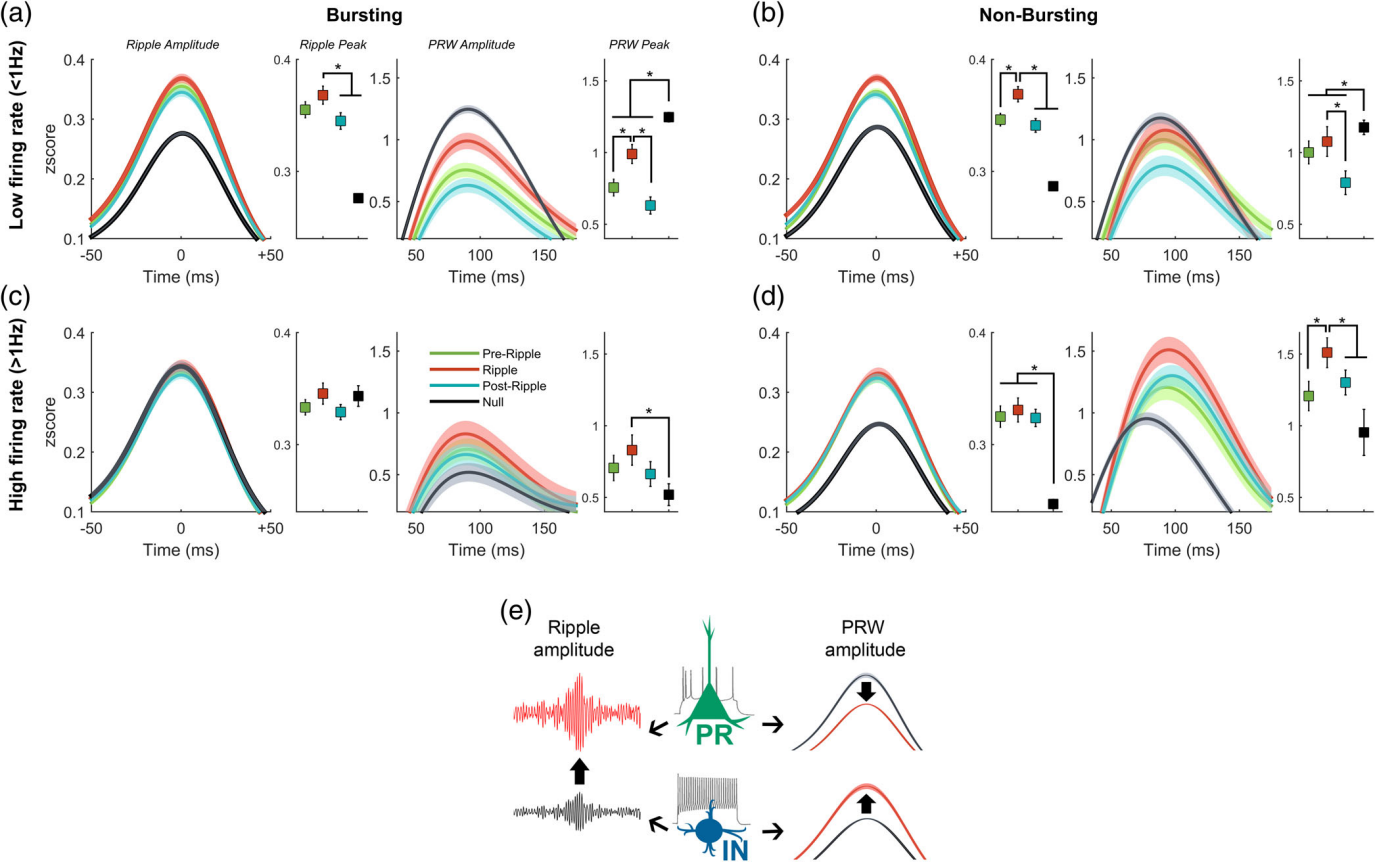
Ripple and PRW amplitudes vary by cell‐type activity and spike‐timing. Mean ± 95% confidence intervals of ripple (left) and PRW (right) envelope amplitudes along with peak values with error bars indicating 95% confidence intervals for principal units (a), nonbursting low‐firing units (b), bursting high‐firing (c) and putative interneurons (d). Schematic of main effects; spikes from principal cells (PR) and interneurons (IN) are associated with greater ripple amplitude, PR spikes are associated with attenuated PRW while IN spikes are associated with enhanced PRW (e). *p < .05

In the earlier analysis of SWR feature changes with behavioral state, the ripple amplitude envelope was greater during quiescence than search (Figure 2b), and larger during remembered compared to forgotten trials (Figure 3b). We therefore sought to examine how spiking in different time windows (preripple, ripple, and postripple) by different cells types affects ripple amplitude (Figure 5). We found that spiking by PR (H[3] = 934.73, *p* = 2.60 × 10^−202^), NBLF (H[3] = 659.32, *p* = 1.39 × 10^−142^), and IN cells (H[3] = 613.21, *p* = 1.38 × 10^−132^) during any period in the 400 ms ripple window was associated with an increase in ripple amplitude (Figure 5a,b,d), whereas spikes from BHF cells had no effect on amplitude (Figure 5c, H[3] = 9.83, *p* = .20; LE‐BHF‐ripple = 0.35z, LE‐BHF‐null = 0.35z, LU had no BHF cells). The contribution of PR and NBLF spiking to ripple amplitude based on spike‐timing followed a similar trend where spiking during the ripple window yielded a larger ripple amplitude compared to the postripple window and null spiking (Figure 5a,b, *p* < .05, Bonferroni post hoc; LE‐PR‐ripple = 0.38z, LE‐PR‐null = 0.25z; LU‐PR‐ripple = 0.41z, LU‐PR‐null = 0.25z; LE‐NBLF‐ripple = 0.37z, LE‐NBLF‐null = 0.33z). With NBLF cells ripple‐window amplitude was also different from preripple spikes (*p* < .05, Bonferroni post hoc). Ripple‐aligned spikes from NBLF cells resulted in the largest ripple amplitude across all cell classes and spike‐times (*p* < .05, Bonferroni post hoc). With IN cells, spikes during the three time‐windows yielded a larger amplitude compared to that seen without IN spiking (Figure 5d, *p* > .05, Bonferroni post hoc).

### 3.7 SUA effects on the amplitude of the PRW envelope

All four cell classes showed differences in PRW amplitude based on spike‐timing; PR (H[3] = 600.57, *p* = 7.59 × 10^−130^), NBLF (H[3] = 111.86, *p* = 4.37 × 10^−24^), BHF (H[3] = 13.20, *p* = 4.20 × 10^−3^) and IN cells (H[3] = 17.98, *p* = 4.0 × 10^−4^). Not surprisingly, the effects on PRW amplitude were similar to those reported earlier on the broadband signal. Spiking by low‐spiking cells (PR and NBLF cells) was associated with smaller PRW amplitudes compared to no spikes (mean LE‐PR‐ripple = 0.96z, LE‐PR‐null = 1.39z, LU‐PR‐ripple = 0.77z, LU‐PR‐null = 1.21z, LE‐NBLF‐ripple = 1.11z, LE‐NBLF‐null = 1.37z, LU‐NBLF‐ripple = 0.94z, LU‐NBLF‐null = 1.17z), whereas spiking by high firing‐rate cells (BHF and IN cells) was associated with larger PRW amplitudes (mean LE‐BHF‐ripple = 1.12z, LE‐BHF‐null = 0.47z; LU had no BHF units; LE‐IN‐ripple = 1.51z, LE‐IN‐null = 0.92z, LU‐IN‐ripple = 1.46z, LU‐IN‐null = 1.12z). In PR cells, null spiking was associated with the largest PRW amplitude, whereas spiking during the ripple resulted in a larger amplitude compared to preripple and postripple spikes (Figure 5a, *p* < .05, Bonferroni post hoc). For NBLF cells, although the pattern was similar to PR cells, the decrease in amplitude due to spiking in the window was not as profound (Figure 5b). Null spiking was associated with a larger PRW amplitude compared to spikes during the ripple, preripple, and postripple, and ripple spikes yielded a larger amplitude than postripple spikes (*p* < .05, Bonferroni post hoc). High firing rate cells had a similar trend to PRW amplitude by spike‐time but with a different direction of magnitude. Spikes during the ripple by BHF cells resulted in larger PRW amplitude compared to no spikes (Figure 5c, *p* < .05, Bonferroni post hoc). But of all cell types, the IN group showed the most striking effects, with spiking during the ripple producing a larger PRW amplitude compared to preripple and no spikes (Figure 5d, *p* < .05, Bonferroni post hoc), as well the largest PRW amplitude compared to all other cell classes and spike times (*p* < .05, Bonferroni post hoc).

### 3.8 Dependency of spiking across ripple time windows

The apparent relationship between spiking in one epoch and LFP/ripple feature in another epoch could in principle be due to joint spiking across epochs, and not to a true time‐lagged modulation. For each unit of each cell type, we calculated the conditional probability of spiking in one time window given a spike from that cell during another window of a ripple event (pre, during, post). Across units from all cell types across all pairs of epochs, a spike in one window typically predicted the *absence* of spikes in the other ripple window. Median probabilities per cell type and window pair ranged from 0 to 0.33. Thus, LFP fluctuations that occur with a lag from the time of spikes do not appear to be an artifact of latent concurrent spiking.

## 4 DISCUSSION

In this study, we showed for the first time in primates that ripple features vary with waking state and memory. By comparing ripple events during quiescent and active periods, we observed that (a) quiescent ripples have larger amplitudes and larger PRWs. Further examination of awake ripples during the memory task revealed that (b) ripples during remembered trials have greater amplitudes compared to forgotten trials, with no change to duration or PRWs. By analyzing ripple‐associated single‐unit activity, we found that (c) ripple amplitude is associated with the activity of low‐firing cells and putative interneurons, whereas the peak and elaboration of the PRW are enhanced by even coarsely timed activity from putative interneurons.

Ripple amplitude is a measure of the magnitude of the high‐frequency ripple oscillation that is thought to reflect both postsynaptic currents and spiking activity by cells within a radius of ~100–200 μm around a recording electrode (Schomburg et al., 2012). The amplitude is dictated by the size and number of active neuronal ensembles that are made up of principal cells and interneurons (Csicsvari et al., 2000), and can be used to predict if similar ensembles are active across ripples (Taxidis et al., 2015).

We classified cells physiologically into four types using burst firing mode and firing rate, although additional functional cell type divisions are possible. All four cell types showed positive modulation of firing rate during ripples, yet only the activity of low‐firing cells and the nonbusting high‐firing cells was associated with increasing ripple amplitude. Low‐firing cells were associated with a decrease in PRW amplitude whereas high‐firing cells showed the opposite effect. Critically, we found that spiking effects on ripple and PRW amplitude were strongest when spikes occurred within the ripple window, yet effects were also observable when spiking occurred within the preripple and postripple periods. This suggests that the effects of spiking on the ripple‐LFP can be extended in time, consistent with previous reports showing similar delayed spike‐LFP relationships (Esghaei et al., 2017). This time‐offset cannot be explained by an increase in the conditional probability of spikes in the preripple or postripple window and spiking within the ripple as we observe that the probability stays the same. The low‐firing cells are likely pyramidal cells, which in the rodent hippocampus are known to display bursting modes (Hemond et al., 2008), with a variable composition across and within subfields (Masukawa, Benardo, & Prince, 1982; Schwartzkroin, 1975). Whereas bursting pyramidal cells have been singled out as necessary for the fast oscillation of ripples (Dzhala & Staley, 2004) and for affecting LFP amplitude (Constantinou et al., 2016), our results suggest that nonbursting principal cells are also strongly associated with the amplitude of the fast ripple oscillation. This positive ripple‐associated modulation of principal cell activity is consistent with previous findings (Csicsvari et al., 1999; Csicsvari, Hirase, Mamiya, & Buzsáki, 2000; Hajos et al., 2013; Klausberger et al., 2003; Klausberger et al., 2004; Le Van Quyen et al., 2008). Most of our spikes and ripples (~92%) were detected on the same electrodes and so we were unable to systematically examine the dependence of the relationship spikes have on the ripple field potential as a function of distance. Although the bundled tetrode arrays used in this study are not ideal for spatial sampling along the septotemporal and transverse hippocampal axes, this is an interesting area for future investigation given the spatiotemporal spread of ripples along the septotemporal axis (Patel, Schomburg, Berenyi, Fujisawa, & Buzsáki, 2013).

The nonbursting high‐firing cell type in our study is likely to contain parvalbumin‐positive interneurons. Parvalbumin‐positive (PV^+^) and bistratified cells show the greatest ripple‐associated increase in spiking rate (Klausberger et al., 2003; Klausberger and Somogyi, 2008), with PV^+^ cells having the greatest excitatory conductance after the ripple peak (Hajos et al., 2013). Axo‐axonic and O‐LM cells typically display negative modulation where they cease to spike during ripples, whereas CCK^+^ interneurons appear to be unmodulated by ripples (Klausberger et al., 2003; Klausberger and Somogyi, 2008). Of the high‐firing cells in our data, we only observed a ripple‐associated positive modulation in spiking (likely due to limited sampling). Perisomatic‐targeting PV^+^ interneurons have been shown to be critical for the initiation of the ripple fast‐oscillation through their recurrent connectivity leading to highly organized inhibition which creates opportunity for synchronous pyramidal cell ensemble activity in CA1/CA3 (Ellender et al., 2010; Schlingloff et al., 2014; Stark et al., 2014; Valero et al., 2015). Pharmacologically blocking perisomatic inhibition on pyramidal cells impairs spontaneous ripple activity and decreases SWR amplitude (Stark et al., 2014; Schlingloff et al., 2014; Gan et al., 2017); moreover, inhibitory conductance in pyramidal neurons during ripples is more dominant than excitatory conductance, correlates with ripple amplitude, and depends on PV^+^ interneurons (Gan et al., 2017). The effects of inhibitory neurons also trail the SWR event, where inhibitory synaptic input leads to the collective afterhyperpolarization of local principal cells following ripples, visible as a postripple deflection in the LFP (English et al., 2014; Hulse et al., 2016). These results are consistent with our finding that spiking of putative PV^+^ interneurons is associated with both larger amplitude ripples and the postripple “inhibitory” wave. The observed increase in ripple amplitude and PRW amplitude during quiescence could therefore be a result of greater PV interneuronal activation in that state compared to during the task. The increased pyramidal‐cell synchrony and larger ensemble activity associated with PV IN ripple activity could form a spatiotemporal “burst” to better propagate efferent signals during sleep, consistent with BOLD responses seen in macaques under anesthesia (Logothetis et al., 2012). Other mechanisms are likely to underlie the differences we observed in waking, for example, during the memory‐guided search.

Waking ripples are increasingly implicated in memory‐guided decision‐making (Jadhav et al., 2012; Papale, Zielinski, Frank, Jadhav, & Redish, 2016; Wu et al., 2017). In rodents, waking ripples contain a higher proportion of co‐activated cell pairs during correct memory recall in a spatial alternation task, suggesting a higher level of coordinated neural activity on remembered trials (Singer et al., 2013). In primates, waking ripples in a visual‐search task occur more frequently and closer to the target during remembered trials suggesting a possible role in memory retrieval (Leonard & Hoffman, 2017). Since the amplitude indexes the size of ripple‐associated ensembles (Csicsvari et al., 2000; Taxidis et al., 2015), it is possible that on average, larger and/or more synchronized ensembles are activated during ripples on remembered trials, though we note that the magnitude of the effects in this study was modest. It is possible that familiar scene stimuli and/or prediction of reward support stronger, more coherent excitatory drive to activate relevant ensembles during the SWR, though determining how such drive modifies ripple magnitude and no other features warrant further study.

## CONFLICT OF INTEREST

The authors have no conflicts of interest.
